# Utility of EBUS-TBNA in PET-positive mediastinal lymph nodes in subjects with extra-thoracic malignancy

**DOI:** 10.1371/journal.pone.0213437

**Published:** 2019-03-11

**Authors:** Ravindra M. Mehta, Pavankumar Biraris, Shekhar Patil, Abhinav Singla, Kumar Kallur, Stefano Gasparini

**Affiliations:** 1 Department of Pulmonary, Critical Care and Sleep Medicine, Apollo Hospitals, Bangalore, India; 2 Department of Medical Oncology, HCG, Bangalore, India; 3 Department of Nuclear Medicine, HCG, Bangalore, India; 4 Department of Biomedical Sciences, Universita Politecnica delle Marche, Ancona, Italy; 5 Department of Public Health, Universita Politecnica delle Marche, Ancona, Italy; 6 Pulmonary Diseases Unit, Azienda “Ospedali Riuniti”, Ancona, Italy; Postgraduate Institute of Medical Education and Research, INDIA

## Abstract

**Background/Aim:**

Patients with primary extra-thoracic malignancy (ETM) often have hyper-metabolic mediastinal lymph nodes (HM-MLN) in the PET-scan done for initial staging or post treatment follow-up. There is scant data on the etiology of HM-MLN in such patients, which can also be due to non-malignant causes. We used endobronchial ultrasound (EBUS) guided sampling to determine the etiology of HM-MLN in patients with ETM and study the relationship between PET-SUV values and a diagnosis of malignancy in this population.

**Materials and methods:**

65 consecutive patients, from March 2013 to March 2017 with either known ETM for primary staging or post-treatment follow-up, with PET CT showing HM-MLN (SUV > 2.5) were included in the study.

**Results:**

65 patients with ETM had EBUS-TBNA for HM-MLN. 20/65 (30.7%) were malignant, 45/65 (69.23%) were benign MLN. In patients with benign etiology of HM-MLN, 6/45 (13.3%) had necrotising granulomatous, 24/45 (53.3%) had non- necrotising granulomatous MLN and 15/45 (33.3%) had reactive MLN. We found discordance (i.e. primary ETM responded to treatment and a new HM-MLN was detected) in 21/65 (32.3%) patients with PET-CT done for initial ETM staging, and 44/65 (67.7%) with a post-treatment PET-CT. showed. Correlating SUV with diagnoses, the SUV values in EBUS-proven malignant MLN were 8.9 ± 4.1, while they were 10.2 ± 5.57 in benign MLN. There was no statistically significant difference between the SUV of benign and malignant MLNs.

**Conclusion:**

This study shows a significant incidence of EBUS-TBNA proven benign diagnoses 45/65 (69.2%) in ‘SUV-deemed-malignant MLN’ and a poor relationship between high SUV and malignant MLN, in patients with known ETM. The ETM related HM-MLN have a significant chance of being benign, and a tissue diagnosis is imperative as it impacts on the treatment plan and prognosis.

## Introduction

Integrated positron emission tomography and computed tomography scans (PET-CT) have revolutionized the diagnostic work-up of malignancy. PET-CT for mediastinal lymph nodes (MLN) is imperative for lung cancer staging and detecting mediastinal spread in extra-thoracic malignancy (ETM).

The approach to PET-positive MLN in the initial diagnosis or follow-up of cancer post-treatment presents certain challenges. In practice, though a low SUV (standardized uptake values) needs tissue confirmation, a high SUV is highly suggestive of malignancy. There are cut-offs of SUV value to differentiate benign and malignant MLN. In general, an SUV exceeding 2.5 in the MLN (hypermetabolic MLN, HM-MLN) is considered highly suggestive of an active process, which has to be clinically correlated to define the risk of malignancy.[1] In lung cancer and PET-positive MLN, SUV values of 2.5[2], 5.2[3] or less are variably described as benign. In ETM, a SUV value of ≥6.3 and above is considered malignant, with sensitivity and specificity of 70.6% and 83.3%, respectively.[4] An accurate tissue diagnoses of PET–positive MLN is vital, for initial staging of primary thoracic or extra-thoracic malignancy or detect recurrence in patients with treated malignancy. However, in PET HM-MLN, there is the confounding possibility of benign disease with inflammatory activity leading to a false-positive high SUV simulating malignancy.[5] The HM-MLN’s in such situations with proven malignancy may not necessarily be malignant, and benign conditions such as tuberculosis or sarcoidosis are possible etiologies of HM-MLN. Though few studies have described this paradox of ‘benign HM-MLN’, this is not adequately studied and the phenomenon of ‘malignant-range SUV–benign MLN’ is not well defined.[6] In tuberculosis endemic regions, SUV’s in benign MLN range from 2.3 to 11.8, and malignant MLN 2.4 to 34, with a statistically significant difference.[6] Another clinical dilemma arises in post-treatment follow-up of malignancy, when the primary tumour regresses or resolves, and HM-MLN’s appear, increase in size or SUV intensity. This clinical disconnect mandates tissue diagnosis of the HM-MLN, to guide management and outline prognosis.

We conducted this study to answer this conundrum in the clinical situation of ETM and HM-MLN. Endobronchial ultrasound (EBUS)-guided transbronchial needle aspiration/biopsy (EBUS-TBNA) has replaced mediastinoscopy as the procedure of choice to sample MLN’s.[7] In patients with primary ETM for initial staging or follow-up of ETM after treatment, we define the etiology of HM-MLN using EBUS-TBNA, and correlation of PET-SUV values with a malignant diagnosis.

## Materials and methods

This was a retrospective analysis of subjects with extra-thoracic malignancy (ETM) who underwent EBUS-TBNA for PET HM-MLN sampling. The study was conducted at a tertiary care referral center between March 2013 and March 2017. Data was analyzed anonymously, and institutional review board clearance (Apollo Hospitals, Bangalore) was waived for this retrospective study, though a procedural informed consent was obtained from all patients. All consecutive subjects with ETM and PET-CT showing HM-MLN (SUV>2.5) were included in the current analysis. The information retrieved from the bronchoscopy database included demographic profile, clinical, radiological and procedure details, pathology and microbiology.

Before the procedure, all patients underwent detailed clinical evaluation, laboratory tests (complete blood count, liver and kidney function tests), chest radiograph and PET-CT. Whole body PET-CT images were obtained using a dedicated 16-slice PET-CT scanner, with 12.23 mCi of FDG contrast; contrast CT images were obtained with 3.75 mm axial slices. SUV values were calculated using body weight, and pathologic metabolic activity foci were evaluated visually and quantitatively. Lymph nodes with a high FDG uptake (SUV >2.5, HM-MLN) were reported as suspicious for metastasis.

The HM-MLN’s were sampled with a convex probe EBUS (7.5 MHz, BF-UC160F; Olympus Corporation, Tokyo, Japan), guided TBNA from various mediastinal and hilar locations under conscious sedation, using midazolam and fentanyl. The EBUS scope was introduced, and the lymph nodes were localised using the endobronchial ultrasound. At least 3 real-time punctures were done in each MLN using a 22-gauge EBUS needle. We made attempts to get both cytological and histological diagnosis with the cell block and core obtained from EBUS sampling. Rapid on-site evaluation (ROSE) was done for all cases. The samples were considered ‘diagnostic’ when a confirmed diagnosis was made, ‘adequate’ if lymphocytes present with or without a conclusive diagnosis and ‘inadequate’ in the absence of lymphocytes or a conclusive diagnosis.

We performed smear for acid-fast bacilli, culture and Xpert MTB/Rif in all cases reported as benign on ROSE.[8] Patients with granulomatous lymphadenopathy were followed up with TB culture, imaging and clinical response. Patients with necrotising granulomatous lymphadenopathy were diagnosed and treated for tuberculosis (TB), if these patients had a positive tuberculin skin test. Patients with non-necrotising granulomatous lymphadenopathy who were negative for tuberculin skin test, TB culture and Xpert MTB/Rif were labelled as sarcoidosis/sarcoid like reaction, and clinically followed with no intervention. HM-MLN with lymphocytes in the TBNA, with all work-up negative, were labelled as reactive MLN, and offered mediastinoscopy or followed clinically.

### Statistical analysis

Statistical analyses were performed using SPSS for Windows, version 21.0. Numeric variations are summarized with mean ± standard deviation and (minimum–maximum) values, while qualitative variations are summarized with numbers and percentages. The comparison of diagnostic accuracies of PET/CT were estimated with a chi-square test. The significant value was P < 0.05.

## Results

There were 65 consecutive patients, with either known primary ETM for staging, or treated ETM for follow-up with PET-CT showing HM-MLN (SUV > 2.5) in the study. The details of the patients are shown in Table 1. An average of two nodes were sampled in each patient, and details of the lymph node locations are mentioned in Table 1. The total number of lymph nodes sampled in the study were 151, with minimum 1 and maximum 4 sampled. Of the 65 ETM patients, 21/65 (32.3%) had a PET-CT showing HM-MLN requiring diagnosis for initial staging, and 44/65 (67.7%) were known cases of ETM for post-treatment evaluation with new HM-MLN’s.

**Table 1:** Details of 65 study patients. (n- no. of samples, Avg.: Average, GIT: Gastrointestinal tract, URT-Upper respiratory tract, MUO- Malignancy of unknown origin).

EBUS–TBNA Cytology (No. of patients)	Benign (n = 45)		Malignant (n = 20)
	Granulomatous (n = 30)	Reactive (n = 15)	
**Characteristics of patients**	Avg. Age: 55.4 years Male /Female 11/19	Avg. Age: 53.33 years Male /Female 12/3	Avg. Age 62.25 years Male /Female 8/12
**Origin of extra-thoracic malignancy** **(No. of patients)**	Breast (7) GIT (4) Genital tract (5) Renal (2) URT (2) Melanoma (3) Hematological (7)	Breast (2) GIT (3) Genital tract (1) Hematological (5) Pancreas (1) Liver (1) Parotid (1) MUO (1)	Breast (6) GIT (4) Genital tract (2) Urinary tract (2) URT (1) Hematological (1) MUO (4)
**Indication for EBUS-TBNA in HS- MLN (No. of patients)**	Primary disease staging (12) Post treatment follow up (18)	Primary disease staging (4) Post treatment follow up (11)	Primary disease staging (5) Post treatment follow up (15)
**Cytopathology Result** **(No. of patients)**	Necrotising granulomatous MLN (6) Non-Necrotising granulomatous MLN [sarcoid like reaction] (24)	Reactive (15)	Adenocarcinoma (10) Poorly differentiated cancer (9) Lymphoma (1)
**Average PET SUV**	12.7 ± 4.8	5.2 ± 3.0	8.9 ± 4.1
**Lymph Node Stations[42]**	Paratracheal (4R and 4L) (26) Subcarinal (7) (20) Other (2R, 2L, 5, 6, 12R, 11R, 11L) (15)	Paratracheal (4R and 4L) (18) Subcarinal (7) (12) Other (2R, 2L, 12R, 11R, 11L) (20)	Paratracheal (4R and 4L) (11) Subcarinal (7) (13) Other (2R, 2L, 12R, 11R, 11L) (16)

Of the 65 patients who had EBUS-TBNA for HM-MLN’s, 20/65 (30.7%) were diagnosed to have malignancy and 45/65 (69.2%) were benign MLN. In the patients with a non-malignancy diagnosis, 6/45 (13.3%) had necrotising, 24/45 (53.3%) had non- necrotising granulomatous MLN and 15/45 (33.3%) had reactive MLN. Of the 24/45 non-necrotising granulomatous MLN (sarcoidosis or sarcoid-like reaction), 10/24 were detected at time of diagnosis or initial staging of the ETM and 14/24 on post-treatment follow up.

**Fig 1:**
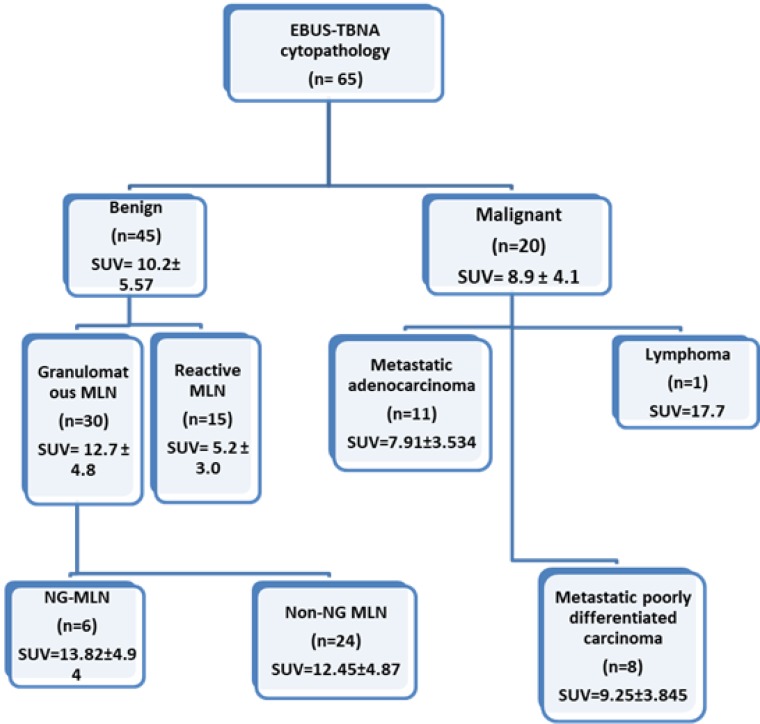
**Results of 65 patients with HM-MLN EBUS-TBNA, with SUV in each group (mean ± SD):** NG MLN: Necrotising granulomatous mediastinal lymph node. Non-NG MLN: Non Necrotising granulomatous mediastinal lymph node.

As shown in Fig 1, the mean ± SD PET-CT SUV value in patients with malignant HM-MLN and benign MLN was 8.9 ± 4.1 and 10.2 ± 5.57, respectively, which was not statistically significant (p = 0.364). Patients in the benign (n = 45) group were further divided into granulomatous (n = 30) and reactive HM-MLN (n = 15). The range of PET-SUV in these subsets were 4.4–17.7, 5.7–26.2 and 2.6–10.7 for malignant, granulomatous and reactive HM-MLN’s respectively. There was a statistically significant difference between PET-SUV of granulomatous (12.7 ± 4.8) and malignant (8.9 ± 4.1) HM-MLN (p = 0.006). There was a statistically significant difference between PET-SUV of granulomatous [12.7 ± 4.8 (mean ± SD)] and reactive [5.2 ± 3.0 (mean ± SD)] HM- MLN (p <0.0001).

Patient with reactive MLN did not consent for mediastinoscopy and were followed up clinically as well as with PET-CT, with an average follow-up of 28.5 months (9–48 months). Of the 15 reactive diagnoses patients, 3 were followed up for 9 months, 3 for 12 months, 4 for 24 months and 5 for 24–48 months. 3 patients had recurrence at sites other than the HM-MLN and died within 12 months.

## Discussion

Approximately 30% of the cases with ETM can metastasize to the mediastinum, manifesting as HM-MLN’s.[9, 10] An accurate diagnosis of PET-CT HM-MLN’s is vital in staging, planning treatment, detecting recurrence and determining prognosis in extra-thoracic malignancies, both primary and post-treatment.

In the diagnostic algorithm of primary ETM or follow-up after treatment, when a HM-MLN shows up on PET, the clinical impression is malignancy. As the specificity of the HM-MLN for malignancy is variable (Table 2), we attempted to define the incidence of benign versus malignant HM-MLN using EBUS-TBNA, to guide management and prognosis. EBUS-TBNA is an effective, safe and accurate MLN sampling modality, however, negative findings may require mediastinoscopy. Yasufuku *et al* reported the sensitivity, specificity, and NPV of EBUS-TBNA in lung cancer as 94.6%, 100%, and 89.5%, respectively, and the accuracy was 96.3%. [7] The data for MLN evaluation by EBUS-TBNA in the specific situation of ETM is limited. We review the utility of PET-CT in detecting MLN spread of malignancy in ETM, and the role of EBUS-TBNA in establishing a definitive tissue diagnosis in these ETM HM-MLN.

**Table 2:** Utility of PET- CT in diagnosis of malignancy with average SUV cut off.

Author (study focus)	No of patients	SUV Cut off	Sensitivity (%)	Specificity (%)	PPV (%)	NPV (%)	Diagnostic accuracy (%)
Lee BE *at al* [3] (Non small cell lung cancer)	110	5.3	81	98	64	99	97
Kandemir *et al* [4] (Pulmonary-extrapulmonary Malignancy)	31	6.3	70.6	83.3	88.9	60	75
Kumar A. *et al*[6] (Study of MLN)	35	6.2	87	70	68	87	77
Song *et al*[12] (Extrapulmonary malignancy)	57	Not defined	81	83	71	82	82
This study	65	6.68	50	26.6	23.26	54.55	33.85

Tournoy *et al* analyzed 92 patients with ETM with suspicion of mediastinal or hilar spread, who had EBUS-TBNA for diagnosis. Majority of the study population (nearly 70%) had head and neck carcinoma, colorectal carcinoma, and renal cell carcinoma. 73% had PET-CT scans and 97% of these showed positive FDG uptake in the MLN. As a final diagnosis, 29 cases (31.5%) had benign conditions (reactive adenopathy, sarcoidosis, silicosis, and hamartoma). Nine patients had a surgical biopsy (mediastinoscopy, video assisted thoracoscopic surgery) which showed metastatic disease. They reported the sensitivity and NPV of EBUS-TBNA in detecting mediastinal spread of ETM as 85% and 76%, respectively.[11]

In a study by Song *et al*., in 57 patients with proven or suspicious ETM, 35 (61.4%) had malignancy and 22 (38.6%) were labelled as benign. EBUS-TBNA identified malignancy in 30 patients. Overall cancer prevalence was 61%. Data about PET/CT scans were available in 49 patients for 71 MLN stations. The sensitivity, specificity, PPV, NPV and accuracy of PET/CT scan per patient were 81%, 83%, 89%, 71%, and 82% respectively. The diagnostic sensitivity, accuracy, and NPV of EBUS-TBNA per patient were 88%, 93%, and 85%.[12]

In another study of EBUS in ETM (n = 48), the sensitivity, specificity, and NPV of EBUS-TBNA for malignancy were 83.3%, 100%, and 90.9%, respectively. When both benign and malignant diseases were considered, the sensitivity, specificity, NPV, and diagnostic accuracy of EBUS-TBNA were 89.2%, 100%, 86.9%, and 93.7%, respectively. 78 LNs were aspirated with EBUS-TBNA in 48 cases with ETM. Results of EBUS-TBNA revealed malignancy in 15 cases (31.2%), tuberculosis in 6 cases (12.5%), sarcoidosis in 4 cases (8.3%), and reactive adenitis in 23 cases (48%). 14/33 cases diagnosed as nonmalignant by EBUS-TBNA underwent mediastinoscopy, and the rest were followed radiologically for at least six months. At the end of the follow-up period, the MLNs diagnosed as sarcoidosis/reactive adenitis by EBUS-TBNA remained stationary, decreased in size or disappeared, and were proven benign.[13]

In our study, EBUS-TBNA revealed malignancy in 20/65 cases (30.76%), and benign diagnoses in 69.24% cases [(tuberculosis in 6/65 (9.23%), sarcoidosis 24/65 (36.92%) and reactive lymph nodes in 15/65 (23.07%)]. All patients with reactive MLN were followed clinically as well as with PET-CT, with no evidence of malignancy developing in the HM-MLN.

In cases with benign diagnoses in this study, 15/45 (33.3%) were primary ETM’s for staging PET-CT, and 30/45 (66.6%) were post-treatment. This is an interesting finding, as in the follow-up ETM cases with benign HM-MLN, PET-CT showed discordance i.e. primary disease responded to treatment and new HM-MLN were detected on surveillance scan. The range of PET-SUV in the subsets were 4.4–17.7, 5.7–26.2 and 2.6–10.7 for malignant, granulomatous and reactive MLN respectively.

There are several explanations for the above observation, namely the significant benign disease prevalence despite high-SUV MLN. Not only active inflammation, but also healed or inactive diseases, such as antecedent or active tuberculosis, silicosis, or anthracosis can result in increased MLN FDG uptake.[14] Various case reports demonstrate the association of tuberculosis and malignancy.[15–17] However data is scant and is limited to case reports, which have suggested the coexistence of TB lymphadenopathy with malignancy,[18–20] due to reactivation/reinfection secondary to the immunocompromised of malignancy/cancer chemo-radiotherapy. This association is very important in countries with endemic TB. The clinical implication of TB is that it can complicate treatment, when surgery or chemotherapy is planned. Other common infective agents besides mycobacteria include toxoplasmosis, fungi, and parasites, which can also evoke a granulomatous response in malignant patients.

Non-infective aetiologies are also described in MLN’s in malignancy. In 1986, Brincker for the first time described an association between systemic sarcoidosis and lymphoma, and used the term “sarcoidosis-lymphoma syndrome” for this association.[21] The association between granulomatous MLN inflammation and malignancy is well described as a ‘sarcoid-like-reaction.[22] This is seen in malignancies such as lung cancer,[23, 24] cutaneous malignancies,[25–27] testicular germ cell tumour,[28–30] renal cell carcinoma,[31, 32] hepatocellular carcinoma,[33–35] and digestive tract cancer.[36, 37] Many hypotheses exist, such as immunological dysfunction related to cancer, or a side effect of cancer therapy. In addition, the phenomenon is described in cancer patients treated with surgery alone, and a theory of "antigenic shedding" from the tumor leading to granuloma formation is postulated.[38–41] There is no agreement on the nomenclature used to describe mediastinal and hilar adenopathy secondary to granulomatous inflammation in malignancy. Various terms exist such as ‘sarcoid like reaction, sarcoid like lymphadenopathy, pulmonary and mediastinal sarcoidosis or simply sarcoidosis’. The clinical relevance of this phenomenon, however, is clear, as no intervention is needed. HM-MLN’s due to this entity need to be recognized, to avoid unnecessary therapy for presumed primary or relapsed malignancy. In our study, these patients were carefully followed-up, with no active intervention.

Our study highlights the high incidence of benign aetiology of HM-MLN’s in patients with ETM, with an additional contribution of tuberculosis in an endemic area. Compared to prior reports, it shows that paradoxically, benign HM-MLN can have significantly higher SUV (10.2 ± 5.57) compared to malignant ones (8.9 ± 4.1). Our study differs from prior reports of PET-CT accuracy in ETM HM-MLN’s, for malignancy with PET-CT showing markedly lower specificities and NPV (Table 2). This study throws light on the variable interpretation of HM-MLN’s in different geographical areas and is a wake-up-call to establish tissue diagnosis in these situations, vitally important to guide management and prognosis.

The limitations of our study include its retrospective single centre nature with a limited sample size, but this compares well with the numbers reported in other studies (31 in the study reported by Kandemir et al).[4] We could not confirm the reactive lymphadenitis diagnoses with mediastinoscopy, as patients were not willing for more invasive surgical procedures, which is a common observation in malignancy situations. Our numbers are skewed towards benign diagnoses, but this is an important feature to highlight in an endemic tuberculosis situation, which is common in many parts of the world.

## Conclusion

In conclusion, this study shows that ‘SUV-deemed-malignant MLN’ or HM-MLN’s on PET-CT have a significant chance of being benign in both primary and post-treatment ETM. This entity of ‘benign HM-MLN’ is more common than mentioned in literature, has important management implications and accurate diagnosis with modalities such as EBUS-TBNA is imperative.

## Supporting information

S1 File PET-EBUS final. Excel sheet for data analysis.
(XLSX)
